# Working Memory and Consciousness: The Current State of Play

**DOI:** 10.3389/fnhum.2018.00078

**Published:** 2018-03-02

**Authors:** Marjan Persuh, Eric LaRock, Jacob Berger

**Affiliations:** ^1^Department of Social Sciences, Human Services and Criminal Justice, Borough of Manhattan Community College, City University of New York, New York, NY, United States; ^2^Department of Philosophy, 751 Mathematics and Science Center, Oakland University, Rochester, MI, United States; ^3^Department of English and Philosophy, Idaho State University, Pocatello, ID, United States

**Keywords:** visual working memory, consciousness, unconscious perception, visual perception, visual awareness

## Abstract

Working memory (WM), an important posit in cognitive science, allows one to temporarily store and manipulate information in the service of ongoing tasks. WM has been traditionally classified as an explicit memory system—that is, as operating on and maintaining only consciously perceived information. Recently, however, several studies have questioned this assumption, purporting to provide evidence for unconscious WM. In this article, we focus on visual working memory (VWM) and critically examine these studies as well as studies of unconscious perception that seem to provide indirect evidence for unconscious WM. Our analysis indicates that current evidence does not support an unconscious WM store, though we offer independent reasons to think that WM may operate on unconsciously perceived information.

## Introduction

Working memory (WM), the capacity to temporarily store and manipulate information in the service of ongoing tasks (Baddeley, 1986), has been correlated with an array of cognitive abilities, including text comprehension, analytical thinking and general intelligence (Fukuda et al., 2010; Johnson et al., 2013). WM, and especially visual working memory (VWM), has also traditionally been linked to perceptual consciousness—that is, it is often assumed to operate on and maintain only consciously perceived information (Baddeley, 1986; Prinz, 2012; Carruthers, 2015).

The goal of this article is to explore whether or not the contents of VWM are invariably conscious. Though some recent studies purport to demonstrate unconscious VWM (Soto et al., 2011; Bergström and Eriksson, 2014), these results have been variously challenged (Prinz, 2012; Carruthers, 2015; Stein et al., 2016). We explore here many of these critiques, as well as several studies not previously discussed, often pursuing different lines of response. Though our analysis likewise indicates that current evidence does not support unconscious VWM, we offer independent reasons to think that WM may operate on unconsciously perceived information.

## Working Memory

A major complicating factor in the debate over the existence of unconscious VWM is that there remains much uncertainty about how best to model the phenomenon of WM, consciously or otherwise. Thus we begin in this section by briefly exploring current models of WM and VWM.

### Current Models of WM

Like both short-term memory (STM) and long-term memory (LTM; Atkinson and Shiffrin, 1968), WM is typically characterized in terms of its functionality. Following the majority of the psychological literature, we define “WM” as the storage system responsible for the maintenance of information in the service of ongoing work—that is, the system that makes available stored information for task-based manipulation—without imposing a limit on its duration or relationship to LTM (Miller et al., 1960; Baddeley, 1986; Luck and Vogel, 2013).

Though perhaps the most influential account of WM is the multicomponent model proposed by Baddeley and Hitch (1974)—which includes two storage systems (a phonological loop and visuospatial sketchpad), a central executive, and more recently an episodic buffer (Baddeley, 2000)—this view has been slowly superseded by more recent state-based models (reviewed in Larocque et al., 2014). Rather than postulating the existence of different systems (buffers) for different memory components, state-based models propose that attention to internal representations such as sensory, motoric, or LTM representations results in different states of information activation. State-based cognitive models have received much experimental support from contemporary cognitive neuroscience.

Cowan (1995), for example, proposes that information in WM exists in one of two states: a capacity-limited state, the so-called “focus of attention” (FoA), or in a capacity-unlimited state called “activated LTM”, which shows temporal decay (see also McElree, 1998; Oberauer, 2002). Such models have been developed to address a set of behavioral findings. For example, Oberauer (2001, 2002) used a retro-cue during a delay period to indicate relevant items from the memory set for the upcoming task. Cued items received attentional prioritization (FoA), whereas uncued items, which were not forgotten, were presumably stored in activated LTM. State-based models dubbed “sensory” or “sensorimotor-recruitment” models have also been developed for perceptual stimuli (Magnussen, 2000; Awh and Jonides, 2001).

Since arguably the most emphasized characteristic of WM is its storage-capacity limit, much work has focused on this aspect of the phenomenon. Two of the most widely cited studies are Miller’s (1956) and Cowan’s (2001), who reported an average capacity of seven items and four items, respectively, for verbal WM. In the visual domain, Luck and Vogel (1997) reported an average capacity of around four individual objects. According to so-called “slot models” of VWM, individual items are stored in a limited number of slots, whereas other items are discarded (Luck and Vogel, 1997). Continuous-resource models, by contrast, treat VWM as highly limited in capacity while allowing the distribution of resources among all items (Ma et al., 2014). According to these models, the number of items remembered is not a fundamental metric, but rather the precision (quality) of memory. A recent variable-precision model further suggests that VWM precision varies from trial to trial and from item to item (Fougnie et al., 2012; van den Berg et al., 2012).

Although it is clear that VWM is limited in capacity, there is currently no agreement about the nature of these limits. Several authors have demonstrated how slot and resource approaches could blend into one another (Souza et al., 2014); it seems likely that a final model, firmly grounded in neural data, will involve aspects of both slot and continuous-resource models (Wolfe, 2014).

In addition, much recent experimentation has attempted to identify the neural underpinnings on WM. Since the discovery of the persistent neuronal activity in monkey prefrontal cortex (PFC) during the delay interval of a WM task (Fuster and Alexander, 1971; Kubota and Niki, 1971) and related findings in human PFC with fMRI (Courtney et al., 1997; Zarahn et al., 1997), it was widely believed that such activity reflects maintenance of WM representations.

This interpretation was, however, questioned when two prominent studies showed that stimulus information during delay periods can be decoded from primary visual cortex with multivariate pattern analysis (MVPA) of fMRI data in the absence of elevated signal levels (Harrison and Tong, 2009; Serences et al., 2009). Furthermore, by using a multiple step retro-cue design to specify the relevant items in a WM task, Lewis-Peacock et al. (2012) showed that MVPA evidence for the non-cued item dropped to the baseline, even though the item could be retrieved by a second retro-cue. These results suggest that persistent neural activity is not necessary to maintain item representations in WM. One prominent idea is that representations are sustained by modification of synaptic weights (Mongillo et al., 2008). A recent study provided converging evidence by using TMS instead of a second retro-cue, to activate memory for the non-cued item (Rose et al., 2016).

Experimental evidence suggests that several neural mechanisms, from intracellular to network based, contribute to WM (for an excellent review see D’Esposito and Postle, 2015). These findings support state-based models, and eliminate the need for specialized buffers. It has been suggested that because persistent neural activity or modulation of synaptic weights is likely a property of most neurons, WM representations arguably can be encoded by neuronal networks virtually anywhere in the brain (D’Esposito and Postle, 2015).

### Varieties of Visual Short-Term Memory

A related obstacle to the study of unconscious VWM is the difficulty in distinguishing its operation from the operation of other visual short-term memory (VSTM) stores. In a well-known study that employed partial report, Sperling (1960) demonstrated the existence of a high-capacity, but limited-duration memory store that he termed “iconic memory”. When post cued, participants were able to report letters from any row of a multi-row letter display. Although this memory store has a high capacity, it decays rapidly on the order of hundreds of milliseconds. According to the classical view, only a few items, selected from iconic memory by attentional mechanisms, form more durable and robust representations that last for several seconds, constituting VWM.

More recently, another type of VSTM has been proposed, so-called “fragile VSTM” (Sligte et al., 2008, 2010), which purportedly has a lower capacity than iconic memory, retains high-resolution representations, and decays linearly over several seconds. According to this proposal, VSTM consists of two limited-duration systems, iconic memory and fragile VSTM, which store many high-resolution representations. These are distinguished from the more robust and durable VWM, which has no duration parameters and stores only one or few high-resolution representations. The existence of fragile VSTM as opposed to mere iconic memory, however, remains controversial (Matsukura and Hollingworth, 2011).

Despite the debate over the fundamental nature of VWM and how it differs from other memory stores, we nonetheless believe that it is possible to assess the current state of evidence for and the possibility of unconscious VWM. In the “Unconscious VWM” section, we explore some of the reasons that theorists have assumed that the contents of VWM are invariably conscious and offer reasons to think that this assumption is questionable.

## Unconscious VWM

Though it is typically assumed that the contents of VWM are always or even must be conscious, the idea that VSTM systems can store unconsciously perceived information for brief durations—on the order of hundreds of milliseconds—is largely uncontroversial (but see Phillips and Block, 2016). Here, we follow most experimentalists working on consciousness by defining visual conscious perception as the subjective experience or visibility of stimuli. Perceptual consciousness can be operationalized (measured) either by objective or subjective measures (Seth et al., 2008).

Researchers have used a variety of experimental paradigms to demonstrate unconscious perception (Kim and Blake, 2005). In a standard masked-priming experiment, for example, stimuli are presented briefly and masked so that they are rendered invisible; yet such stimuli are nonetheless thought to be perceived unconsciously because they *prime* or affect downstream behavioral responses (Kouider and Dehaene, 2007). In some experimental paradigms, stimuli are masked and presented for longer durations (Tsuchiya and Koch, 2005; Persuh et al., 2016). Some type of memory store is implicated in such studies, as behavioral responses are performed in the absence of the perceived objects. We have strong experimental evidence for unconscious response inhibition, a form of cognitive control (van Gaal et al., 2008, 2010, 2012) and for unconsciously deployed metacognitive judgments (Charles et al., 2013). These are higher order cognitive functions, closely associated with WM. Recently, direct evidence for unconscious iconic memory storage has been provided (Sergent et al., 2013; Thibault et al., 2016; Xia et al., 2016). Why, then, do so many assume a link between consciousness and VWM?

### Associating Consciousness and VWM

Perhaps the central reason for this assumption is that many maintain that there is a commonsense tie between WM and consciousness. Stein et al. (2016), for example, write that:

*WM corresponds well to our everyday phenomenology of “keeping in mind” some information over a short period of time. From this phenomenology, it seems clear that WM is intricately interwoven with conscious awareness. It is difficult to imagine a situation in which we are not consciously aware of the stimuli that enter WM (p. 1)*.

That many assume a folk-psychological connection between consciousness and WM is consistent with the long history within consciousness studies of assuming that many or even all high-level mental activity requires consciousness (for review see Rosenthal, 2008; Shea and Frith, 2016).

Unsurprisingly, then, many models of consciousness or WM implicitly build one phenomenon into the other. Baddeley (2000), for example, modified his original multicomponent model to include an episodic-buffer, which he conceives of as acting as a global workspace (GWS) in Baars’s (1988) terminology. The GWS is purportedly a central neural module, which enjoys long-range connections to many areas of the brain; it is thus capable of making information encoded in it available for widespread impact on many neural functions and behavior. According to GWS theories of consciousness (Baars’s, 1988; Dehaene and Naccache, 2001), the representations in the GWS determine the contents of consciousness. Much neuroimaging data supports GWS theories, purportedly showing that the difference between conscious and unconscious perception consists in differing activations of frontal/parietal areas and widespread connections to other areas (but see Siclari et al., 2017). Although on Baars’s (1997) own view the contents of WM need not necessarily be conscious, on Baddeley’s GWS-based model of WM, the contents of VWM are invariably conscious (see Carruthers, 2015).

Similarly, Prinz’s (2000, 2012) attended intermediate-level representation theory of consciousness identifies the contents of consciousness with appropriately attended representations at the intermediate level inspired by Marr’s (1982) and Jackendoff’s (1987) models of the visual system, and since Prinz equates the relevant kind of attention with the gateway to WM, he likewise holds that only those representations that are available to WM are conscious. Such accounts thereby rule out the unconscious operation of VWM. There are, however, many theoretical reasons to be skeptical that VWM must take as input consciously perceived information only.

### Dissociating Consciousness and VWM

First, we note that the functional characterization of VWM offered at the outset—that is, a limited-capacity system that allows one to store and manipulate information—is theoretically neutral insofar as it does not invoke consciousness. If this characterization of VWM is fair, then it remains an open experimental question whether unconsciously perceived information can be encoded in VWM.

Indeed, it is far from obvious that folk psychology includes a tie between WM and consciousness. Since common sense admits of unconscious perceptual states, it would seem at least open that it also includes the possibility that we can “keep in mind” unconsciously perceived information over a short time. Even if Stein, Kaiser and Hessemann were correct that it is somewhat difficult to generate clear examples of such events, it may simply be because such encoding in VWM is unconscious—and so we are unaware from the first-person perspective that this encoding is occurring.

The assumption that higher mental functions require consciousness is increasingly suspect (Shea and Frith, 2016), and many independently motivated accounts of consciousness do not involve any assumptions about the nature of the contents available to WM. For example, Tononi’s (2004) integrated-information theory, according to which mental states are conscious just in case they reach a suitable level of information integration, does not theoretically require that visual contents encoded in VWM exceed the relevant threshold of integration. Likewise, higher-order theories of consciousness, according to which a mental state is conscious just in case one is suitably aware of oneself as being in it (Armstrong, 1968; Rosenthal, 2005), provide no reason to think that we must be so aware of the contents that are made available to VWM. These theories of consciousness have experimental support (Lau and Rosenthal, 2011; Tononi et al., 2016) and are consistent with unconscious VWM.

With the development of state-based models of WM, the link between consciousness and WM can be reformulated by asking whether a specific WM state only takes as input conscious information. It seems likely that nonattended information in activated LTM can be represented unconsciously. It has been suggested that perhaps different states of WM correspond to conscious (FoA) and unconscious (activated LTM) information, rather than attended and unattended information (Silvanto, 2017). It might seem obvious that information in the FoA should always be conscious, but that is not necessarily so, as we can attend to unconscious stimuli (Norman et al., 2013).

The possibility of unconscious VWM is thus interesting in at least two ways. First, it remains an independently interesting question to determine what, if any, mental functions must occur consciously (Berger, 2014). Second, convincing evidence of unconscious WM would require reevaluation and perhaps even rejection of some theories of consciousness.

## Current Experimental Evidence for Unconscious VWM

Several studies have attempted to demonstrate unconscious VWM.

### Unconscious Operation of VWM

In perhaps the earliest explicit attempt to demonstrate the unconscious operation of VWM, Hassin et al. (2009) presented participants with a rapid series of disks and participants were required to press a button to indicate whether the disks were filled or unfilled. Unbeknownst to the participants, in some conditions the series of disks formed a pattern, which would indicate whether or not a forthcoming disk would be filled. Although participants in the pattern conditions were not able to report on these patterns, they were faster at determining whether the disks were filled than in the non-pattern conditions. Hassin et al. (2009) argue that this task required the unconscious operation of VWM because it was necessary for participants to hold in memory a series of disks and compare them to visible disks to determine whether or not they formed a pattern, even though participants did not consciously hold or compare the disks in memory.

Proponents of the view that the contents of VWM are necessarily conscious have offered various critiques of this study. Prinz (2012, p. 96), for example, proposes reasonably that since the stimuli are quite complex, they likely outstrip the limited capacity of VWM and instead implicate fragile VSTM (see Carruthers, 2015, p. 86).

But whether or not these stimuli engage only VSTM, it is crucial to note that in all five of these experiments the stimuli were fully visible. Thus, as Carruthers (2015, p. 86) observes, such results arguably demonstrate only that the computed pattern and the resultant expectations are not among the contents of WM, and that this does not show that VWM encodes unconsciously perceived information, and similar considerations likewise undermine what may seem to be evidence of unconscious VWM from experiments involving implicit change detection. Several studies have reported unconscious change detection: for example, studies have found that implicit change detection in the orientation of an item influenced performance on subsequent orientation-judgment tasks (Fernandez-Duque and Thornton, 2000). At first sight, this might seem like *de facto* evidence of unconscious VWM. However, stimuli in these tasks were also fully visible and the delay between two displays was only 250 ms, which arguably is an interval that taps into other types of VSTM rather than VWM.

In other words, reflection on this work reveals an important distinction in types of studies of unconscious VWM. First, there is the question of whether or not the manipulations of VWM content require consciousness—what we call henceforth the “unconscious operation” of VWM. This is, however, distinct from the question of whether or not unconsciously perceived information can be encoded in VWM. This latter question is our central interest here.

Other recent studies have likewise provided evidence for the unconscious *operation* of VWM, albeit more indirectly (Bona et al., 2013; Bona and Silvanto, 2014). Bona et al. (2013), for example, examined the relationship between performance and conscious experience in VSTM task. A memory cue was followed, after a delay, by a probe stimulus and participants reported the orientation of probe relative to the memory cue in a forced-choice procedure. After performing a discrimination task, participants reported their conscious experience of the cue stimulus. On half of the trials, masked distractors were presented during the delay period and participants also rated their conscious experience of distractors. Data from this study revealed a double dissociation between performance and conscious experience. Discrimination performance was negatively affected only when distractors differed significantly from cue orientation, regardless of distractor visibility. Cue visibility showed the opposite pattern: visibility was unaffected by distractor orientation. Cue visibility ratings were, however, lower for invisible distractors. These results led authors to conclude that the VSTM memory trace, on which performance is based, is different from the content of conscious experience of VSTM. Furthermore, this evidence led to the proposal of separate representation for conscious experience, a so-called “conscious copy” model of WM introspection (Jacobs and Silvanto, 2015).

Although cue stimuli in VSTM task in Bona et al. (2013) were fully visible and thus cannot directly reveal whether or not unconscious content of VWM is possible, a double dissociation, if independently confirmed, would nonetheless provide indirect evidence for the unconscious operation of VWM. But a simpler explanation of their results would be as follows. The distractor images modified the cue memory representations—with larger deviations in distractor orientations causing larger shifts in cue memory representations—which in turn affected forced-choice discrimination performance while leaving the vividness of the conscious experience of cue memory intact. In other words, one’s conscious experience of a cue is different for different distractor orientations, but equally vivid; one can represent different orientations, equally vividly. It is also possible that invisible distractors modify the vividness of cue memory without affecting forced-choice discrimination performance. Even with lower visibility, participants might have had enough orientation information to sustain performance. This reasoning would explain double dissociation results without invoking an additional “conscious copy” representation in WM.

We turn in the “Unconscious Content of VWM” section to studies that more directly assess the question of whether or not unconsciously perceived information can make it into VWM.

### Unconscious Content of VWM

In a series of four experiments, Soto et al. (2011) presented participants with a masked Gabor patch, to prevent the patch from being consciously perceived. Participants were instructed to keep this cue in memory. After a delay of several seconds, a second Gabor patch, the target, was presented. Participants were then asked to perform cue-target orientation discrimination and to report their awareness of the cue on a scale from 1 to 4, with 1 indicating no visibility. In some experiments, a distractor was presented after the cue or participants were presented with two cues. In all four experiments, orientation discrimination was above chance level (50%) for trials with visibility ratings of 1.

The authors suggested that data support the existence of unconscious VWM rather than a mere priming effect mainly due to the above-chance performance despite the presence of a distractor and because the gap between cue and target was 5 s in some experiments. These factors purport to show that the cues were held in memory during an ongoing task, and so in VWM.

Some critics of unconscious VWM allege that these results can be explained without appeal to it. Prinz (2012, p. 86), for example, urges that the fact that Soto and colleagues did not find any decrease in performance even after delays of up to 5 s suggests that fragile VSTM, and not VWM, is implicated insofar as VWM putatively shows signs of decay at around 4 s (Zhang and Luck, 2009). By contrast, Carruthers (2015, p. 87) proposes that deploying attention to unconsciously perceived stimuli might increase the processing of that signal without requiring that the stimuli be encoded in VWM. That is, one might urge, *contra* the authors, that even though participants held the cue in mind over a delay and performed a distractor task, since the information was not in any way manipulated, participants’ increased performance was a mere priming effect, requiring storage only in VSTM. Unlike STM, which involves only storage, WM involves not only the maintenance, but also the potential *manipulation* of information (Baddeley, 1986; Luck and Vogel, 2013).

As Stein et al. (2016, p. 2) observe, however, a more pressing problem for these experiments is that they depended upon verbal reports of cue visibility. Although such subjective measures are often thought to better reflect perceptual consciousness than objective ones such as forced-choice discrimination (Merikle et al., 2001; Dehaene and Changeux, 2011), it is well known that such reports are prone to response bias (Schmidt, 2015; Peters et al., 2016). In other words, trials rated 1 (no visibility) might reflect weak conscious perception of cues, which participants simply fail to report because of conservative standards for regarding a stimulus as seen. Supplementary data demonstrate that participants reported conscious perception of the cue on roughly half of the trials. Thus supposing, for a hypothetical experiment, that participants reported conscious perception on a large majority (e.g., >90%) of trials, only 10% of trials would be analyzed. Clearly such evidence of unconscious VWM would be met with skepticism.

For that reason, objective measures of perception, such as *d*′ (the signal-to-noise ratio; MacMillan and Creelman, 2005), are typically preferred. Although Soto et al. (2011, p. R912) reported *d*′, they based their calculation only on trials with a rating of 1 and thus, as noted by Stein et al. (2016, p. 2), their reported pseudo-d′ is not a bias-free measure. Although in their reply to Stein et al. (2016), Soto and Silvanto (2016) report the actual *d*′, it is not clear whether it is statistically significant.

It is also important to note that most studies of unconscious VWM implicitly assume a slot model and that, according to continuous-resource models, the fundamental metric is not the number of objects stored but instead a precision of each representation, which can vary between items and between trials. It is thus more plausible to suggest that low (although above chance) performance in Soto and colleagues’ study stems from the noisy encoding of cues, which were presented briefly and then masked.

Although not discussed by Stein et al. (2016), more recent studies (Bergström and Eriksson, 2014, 2015; Dutta et al., 2014; King et al., 2016; Trübutschek et al., 2017) have employed similar approaches and thus suffer from the same concerns about subjective measures of stimulus visibility. Trübutschek et al. (2017), for example, used a masking paradigm to render stimuli invisible in a spatial delayed-response task and collected behavioral as well as magnetoencephalography (MEG) data in perception and WM paradigms. Their study set out to address two major concerns facing previous studies of unconscious WM: (1) participants in the previous studies could have erroneously reported weakly perceived targets as unseen (the “miscategorization hypothesis”); and (2) participants could have made immediate guesses about the target and maintain these guesses in conscious WM (the “conscious maintenance hypothesis”). Both hypotheses suggest that the results of previous studies could be due to conscious WM. To test these hypotheses, Trübutschek et al. (2017) examined event-related fields, performed time-frequency analyses, and used machine-learning approaches to dissect neural activity on seen and unseen trials. Importantly, if the miscategorization or conscious maintenance hypotheses were correct, neural signatures on unseen correct trials would resemble neural signatures on seen trials. The location of subjectively unseen targets was reported above chance, seemingly confirming earlier reports of unconscious WM (Soto et al., 2011). Both conscious perception and conscious WM showed shared brain signatures; classifiers trained to separate unseen and seen trials were able to generalize from one task to the other. Furthermore, conscious perception and conscious WM were characterized by sustained desynchronization in the alpha/beta band over frontal cortex and a decodable representation of target location in posterior cortex. Importantly, such activity was not demonstrated for targets on unseen correct trials and classifier generalization was unsuccessful. These results provide evidence for unconscious WM, possibly suggesting that synaptic mechanisms support unconscious WM (Mongillo et al., 2008).

One recent model of unconscious WM supported by synaptic mechanisms suggests that WM does not implicate attention, but instead distinct states of WM possibly representing conscious and unconscious information (Silvanto, 2017). According to the model, retro-cues (Oberauer, 2001) or TMS pulse (Rose et al., 2016) may bring non-cued WM content to conscious experience. This model is consistent with findings showing that we can attend to unconscious information (Norman et al., 2013) and that items in unconscious WM are resistant to distractor interference, which requires attention. This is certainly an interesting possibility that awaits confirmation.

Trübutschek et al. (2017) note that one of the criteria for WM is manipulation of stored information (Baddeley, 1986; Luck and Vogel, 2013) and that the content of putative unconscious WM was not manipulated in their study. However, the major problem with their study is that, as in previous studies of unconscious WM, consciousness was measured using subjective reports. Although MEG evidence showed desynchronization in the alpha/beta band only for seen trials and not for unseen correct trials, the masking procedure can create a variety of visual experiences that do not necessarily map onto response options. For example, for briefly presented targets in studies using metacontrast masking, a target might change the appearance (e.g., brightness) of the mask, without being perceived (Bachmann and Francis, 2014). In such a case, participants would have some location information in the absence of the conscious perception of the target, supporting above-chance performance on subjectively unseen trials without unconscious WM. If this possibility could be ruled out, we would have a strong evidence for unconscious WM.

Some studies of unconscious WM have, however, not relied on subjective measures of stimuli invisibility. Using the method of the breaking of continuous-flash suppression (CFS), Pan et al. (2014) reported biasing of visual perception by cues held in unconscious VWM. In CFS, a rapidly changing pattern of Mondrian patterns presented to one eye suppresses conscious perception of stimuli presented to the other eye for several seconds (Tsuchiya and Koch, 2005). Pan et al. (2014) instructed participants to hold in memory a face cue, which was rendered unconscious by a pattern mask. Signal-detection analysis showed that *d*′ was not significantly different from zero. Using CFS to suppress target face processing, the contrast of the target face in the suppressed eye was gradually increased until participants consciously perceived the face and reported its location. Interestingly, when the target face matched the initial face cue held in VWM, the participants’ reaction times were quicker. In a series of control experiments, the authors showed that this effect occurs only when the memory cue is maintained in VWM.

Although Pan et al. (2014) did report that the objective measure of consciousness, *d*′, showed that stimuli were not consciously perceived, their experiments did not require participants to manipulate the remembered information in any way. In previous studies (Soto et al., 2011), participants compared the orientation of a stimulus putatively held in unconscious VWM to a target stimulus. In this study, by contrast, unconscious information simply influenced visual processing. Although some type of memory was clearly involved, this experiment is thereby subject to the kind of criticism that Prinz leveled at Hassin et al. (2009) study: that the storage does not clearly meet the minimal requirements of VWM, which involve not only the maintenance, but also the manipulation of information.

Recently, Bergström and Eriksson (2017), conducted an fMRI study and used objective measure to assess participants’ awareness of memory items suppressed with CFS. Participants performed a delayed match-to-sample task in three conditions: a baseline condition with a CFS mask only, a conscious condition with objects only, and an unconscious condition in which objects were suppressed with CFS. Participants were first tested in the pre-fMRI session with a 5 s delay period and then in an fMRI session with a 5–15 s delay period. On each trial, participants first performed a recognition task, followed by YES/NO detection response, and finally they rated their visual experience on perceptual awareness scale. Only trials with a rating of 1 (no perceptual experience) on the scale were selected for the analysis of the unconscious condition. Although memory performance (*d*′) on the recognition and detection tasks was above chance during the pre-fMRI session, neither was better than chance during the fMRI session. Multivariate pattern analyses of fMRI data from the unconscious condition could classify presence vs. absence of memory items in prefrontal and occipital cortex, demonstrating the maintenance of unconsciously presented memory items. The authors further suggested that maintenance of unconscious representations in their study depended on persistent neural activity, above activity-silent synaptic changes, and the results are therefore inconsistent with the model of unconscious WM proposed by Trübutschek et al. (2017).

One difficulty with this study is that we have no behavioral evidence for the maintenance and manipulation of information, which figures in the operational definition of WM. Although the authors used objective measures of awareness and showed that behavioral performance (*d*′) was at chance, the delay period during the fMRI session was long (5–15 s), and likely contributed to decrease in performance; the authors acknowledged that this and other factors related to fatigue could have played a role in explaining performance. In future experiments, the authors could chose to include a set of randomly intermixed trials on which performance is assessed immediately after the presentation of stimuli.

A set of studies by Rosenthal and colleagues (Rosenthal et al., 2010, 2016; Rosenthal and Soto, 2016) on learning higher-order visuospatial sequences in the absence of perceptual awareness provides additional evidence relevant for understanding the relationship between consciousness and WM. The authors used a dichoptic masking protocol to prevent conscious perception of a complex second-order visuospatial sequence, which was presented repeatedly during the learning phase of the experiment across four monocular locations. Participants were then prompted to discriminate old from new sequences and to rate their confidence during the recognition phase of experiment that followed 20 min later. A control experiment revealed that participants were at chance in reporting the eye of origin for individual sequence stimuli. Although participants were at chance at discriminating old from new sequences, confidence ratings revealed that learning did occur. Recognition memory was associated with V1 activity, as a part of a network that included the hippocampus. Because the learning of visuospatial sequences requires maintenance and manipulation of information over several seconds, thereby meeting the operational definition of WM, these results seem to provide evidence for unconscious WM. Furthermore, because recognition memory, that was associated with hippocampal activation, was probed after a significant delay, these results also reinforce a strong connection between WM and LTM systems.

Two issues related to these findings are worth mentioning. First, the visuospatial sequences were repeatedly presented to participants and tested for recognition 20 min later. This methodology differs from typical WM tasks in which target stimuli are presented only once and tested for recognition several seconds later. Repeated presentation of stimuli arguably can induce learning through mechanisms independent of WM. The second issue concerns the measurement of recognition memory. Here, the authors showed that both accuracy and sensitivity (*d*′) analyses showed no evidence of learning. It was the usage of metacognitive measures, confidence ratings and type-2 sensitivity, that demonstrated learning. The relationship between perceptual consciousness and metacognitive awareness is not well understood, however, and some recent studies suggest that metacognitive judgement is possible in the absence of perceptual sensitivity or awareness (Charles et al., 2013; Scott et al., 2014; Jachs et al., 2015).

A recent study by Samaha et al. (2016) examined the relationship between metacognitive awareness and WM. Participants performed perceptual and WM tasks for stimuli that were matched for performance (*d*′), but that varied in confidence ratings (metacognitive awareness). If WM depends on consciousness, then on trials with higher confidence ratings WM performance should improve. This hypothesis naturally depends on the assumption that metacognitive awareness is a good measure of perceptual consciousness. But the authors found no evidence for this hypothesis, suggesting that WM is independent of conscious perception. Although these results are suggestive, it should be noted that one does need an additional theoretical assumption to conclude that WM can store unconsciously perceived items. Some minimal perceptual consciousness of to-be-remembered items might be required for WM, even if further increase in metacognitive awareness does not improve WM performance.

An important issue raised by studies that employ metacognitive judgments concerns the relationship between perceptual consciousness and metacognitive awareness. Jachs et al. (2015) have demonstrated that whereas stimulus awareness has a strong effect on perceptual discrimination (*d*′), the effect on confidence judgments (metacognition) was much weaker. These results suggest that perceptual and metacognitive judgments do not operate on the same input and that metacognition is not tightly linked to perceptual consciousness. Converging evidence comes from studies on error detection. Charles et al. (2013) showed that participants were able to detect their errors above chance even under subliminal conditions. They proposed that two distinct mechanisms exist for metacognitive judgments, conscious and unconscious evaluation systems. Corroborating evidence from a recent study that evaluated error-monitoring performance in schizophrenia patients and healthy controls showed that only conscious metacognition is affected in schizophrenia, whereas unconscious monitoring performance remained intact (Charles et al., 2017). These results are important because they suggest that: (1) if metacognition can be dissociated from perceptual awareness, then we cannot use metacognitive judgments to assess perceptual consciousness; and (2) since metacognition is considered a higher-order process, it strengthens the evidence that higher-order processes related to WM can be deployed unconsciously.

## Ways Forward in the Study of Unconscious VWM

Having examined the current literature regarding the relationship between consciousness and VWM, we find that there is no definitive evidence for unconscious VWM. But we see no in principle barrier to the demonstration of unconscious VWM. Thus in closing we offer recommendations for how to move forward in the study of unconscious VWM, in light of the lessons gleaned from the work that has already been done.

In short, because of the problem with response bias, proper experimental design should use forced-choice discrimination as a measure of consciousness and an indirect measure to demonstrate storage and manipulation of information.

Moreover, in order to study the unconscious content of VWM, and not merely VWM’s unconscious operation, target stimuli must of course somehow be invisible to visual consciousness. But one well-known pitfall for studies that involve the technique of visual masking is that it typically involves brief presentation times and masks that inevitably degrade the stimuli, thereby reducing their signal strength. Consequently, some theorists have speculated that the fact that we have not decisively experimentally demonstrated many higher cognitive functions occurring unconsciously may be an artifact of our current methods for masking stimuli (e.g., Lau, 2009; Persuh et al., 2016). Thus, were a legitimate study of unconscious VWM involving masking to be devised, the failure of participants to successfully perform the memory task might be explained not by the fact that the unconsciously information cannot be encoded in VWM, but by the fact that unconscious perception, as it is currently studied, is typically weak. New experimental techniques might be developed to explore the full extent of unconscious perception and unconscious VWM (Tsuchiya and Koch, 2005; Persuh et al., 2016).

For these reasons, blindsight—wherein people with cortical damage to V1 are capable of discriminating stimuli in their blind regions (scotoma) though they report no perceptual consciousness (Weiskrantz, 1986)—may seem to provide a particularly promising route for studying unconscious VWM. In blindsight patients, high contrast stimuli can be presented for unlimited durations. Skeptics regarding unconscious VWM might regard blindsight responses as drawing on a type of fast and automatic processing akin to priming, but most participants in studies of blindsight are not required to make speeded responses; instead they have several seconds to respond to visibility and forced-choice discrimination questions. To respond correctly, unconscious visual information must be stored and VWM seems a likely candidate. To our knowledge, however, no study of blindsight has utilized a standard task for testing VWM, such as requiring participants to manipulate unconsciously perceived information after a sizable delay period.

We recognize, however, that there are several criticisms of blindsight, such as the fact that it arguably involves a form of degraded normal vision (Weiskrantz, 2009) or that a participant might unconsciously perceive the stimulus, on that basis make a conscious guess, and then retain the conscious representation in memory before responding (Stein et al., 2016). These criticisms can certainly be addressed with proper experimental design. Although blindsight is a paradigmatic example of a dissociation between objective and subjective measures of consciousness, a modified experimental design could employ a combination of objective measure of consciousness and an indirect measure of storage and manipulation of information.

One might, however, prefer evidence of unconscious VWM in healthy individuals. Lau and Passingham (2006) coined the expression “relative blindsight” to describe differing levels of subjective visibility for stimuli in healthy individuals that were nonetheless matched for objective task performance. It has been proposed that this approach could be a fruitful way to study unconscious WM (Samaha, 2015). Although the original study faced several criticisms (Jannati and Di Lollo, 2012; Balsdon and Azzopardi, 2015), which were addressed in subsequent studies (Maniscalco et al., 2016; Peters et al., 2016), the proponents of the relative blindsight paradigm report that they have abandoned this strategy due to very small, although reproducible effects (Peters et al., 2016). Relative blindsight might thus not be a particularly fruitful avenue for future studies of unconscious WM. A related concern is that relative blindsight cannot provide direct evidence for unconscious WM, because we cannot exclude the possibility that some level of perceptual consciousness is necessary for WM, even if WM performance is not modulated by further changes in perceptual consciousness.

Some studies with healthy participants employing CFS, by contrast, may have provided indirect evidence for unconscious VWM. Sklar et al. (2012), for example, presented participants with an equation masked by CSF for up to 2 s and then asked them to verbalize a visible number. When the result of the equation and the number were congruent participants were faster. Individual participants that performed above chance based on binomial distribution analysis were excluded from the analyses. Since recent evidence suggests that VWM is engaged not only for maintenance of visual information but also for items still in view (Tsubomi et al., 2013), these results arguably suggest the existence of unconscious VWM. There is, however, some concern about the replicability of this study (Karpinski et al., 2017). A more definitive study of unconscious VWM in healthy individuals might use a similar CFS-based set-up but use a more traditional task regarding VWM.

Perhaps more pressingly, given the present lack of consensus regarding the nature of WM and its neural basis, it would be reasonable for future studies of unconscious VWM to use set sizes and delay intervals between the presentation of a to-be-remembered stimulus and the memory task that clearly separates VWM from other memory systems such as fragile VSTM.

Lastly, we take it that perhaps the *key* feature of WM, which distinguishes it from STM and LTM, is that information encoded in WM is available for use in ongoing tasks. That is, such information must be available for *manipulation*. To be clear, we do not claim that information stored within VWM must be manipulated; it is consistent with current models that such information may simply be stored and forgotten. But, to count as being encoded in VWM, information must at least be disposed to be manipulated. Many purported studies of unconscious VWM that do not involve information manipulation are open to the criticism that the information gleaned from unconsciously perceived targets merely primes participants or is stored in (fragile) VSTM (Soto et al., 2011). Perhaps the manipulation of target information might involve participants’ being primed after mentally rotating remembered stimuli (Hyun and Luck, 2007) or after performing arithmetic operations on remembered numbers (see Sklar et al., 2012).

Whether or not there are ways to modify standard paradigms for studying WM, any required manipulation of target information will doubtless increase the difficulty of such tasks, thus reducing the likelihood that participants can successfully perform them. Coupling this with the problem that unconscious stimuli are often weakly encoded, the possibility that unconsciously perceived information could survive the relevant delay period to be manipulated may seem remote. But so far as we see it, there is nothing theoretically that would rule out the possibility of unconscious VWM.

## Author Contributions

MP developed the manuscript concept. MP, EL and JB wrote the manuscript and approved the final version of the manuscript for submission.

## Conflict of Interest Statement

The authors declare that the research was conducted in the absence of any commercial or financial relationships that could be construed as a potential conflict of interest.
